# Supplementary material for: Differential impacts of germline and adult aggrecan knockout in PV+ neurons on perineuronal nets and PV+ neuronal function
Source: Mol Psychiatry. 2025 Jan 22;30(7):2907–21. doi: 10.1038/s41380-025-02894-5 (PMC12185343; doi:10.1038/s41380-025-02894-5)
Supplement: Supplementary file 1 — S1 [file 41380_2025_2894_MOESM1_ESM.docx]

**Table S1, qPCR primers.**

| **Gene name** | **Forward 5’-** | **Reverse 5’-** |
| --- | --- | --- |
| ACAN (exon 4) | GCTTGCCTACAGAACAGCGCCA | GGGGCGTGTGGATGGGGTATCT |
| BCAN | CTCGGCGGCTATGAGCAGTGTG | CAGGCCTCTCGTGGGTTCTGGA |
| VCAN | TGGCCCAGAACGGAAATATCA | ACTAGCCCGGAGTTTGACCAT |
| NCAN | ACGCCTACTGCTTCCGAGCTCA | GGAGGCCCCTCTGCTGACACAA |
| TNR | CCTTGCTGCGAGACCAGTGCAA | TAAAGTTGCCATGGCCGCTGCA |
| SEMA3A | GGCTGGTTCACTGGGATTG | CCGTTTGCATAGTTTGCTCTGG |
| GAPDH | AGGTCGGTGTGAACGGATTTG | TGTAGACCATGTAGTTGAGGTCA |
| PPIA | TCCGACTGTGGACAGCTCTA | ATTGCGAGCAGATGGGGTAG |
