# Supplementary material for: Differential impacts of germline and adult aggrecan knockout in PV+ neurons on perineuronal nets and PV+ neuronal function
Source: Mol Psychiatry. 2025 Jan 22;30(7):2907–21. doi: 10.1038/s41380-025-02894-5 (PMC12185343; doi:10.1038/s41380-025-02894-5)
Supplement: Supplementary file 2 — Supplementary materials [file 41380_2025_2894_MOESM2_ESM.docx]

**Supplementary Data to Grødem, Thompson et al.**

**“**Differential Impacts of Germline and Adult Aggrecan Knockout in PV+ Neurons on Perineuronal Nets and PV+ Neuronal Function**”**

**This document contains supplementary figure S1-S6, and Table S1**


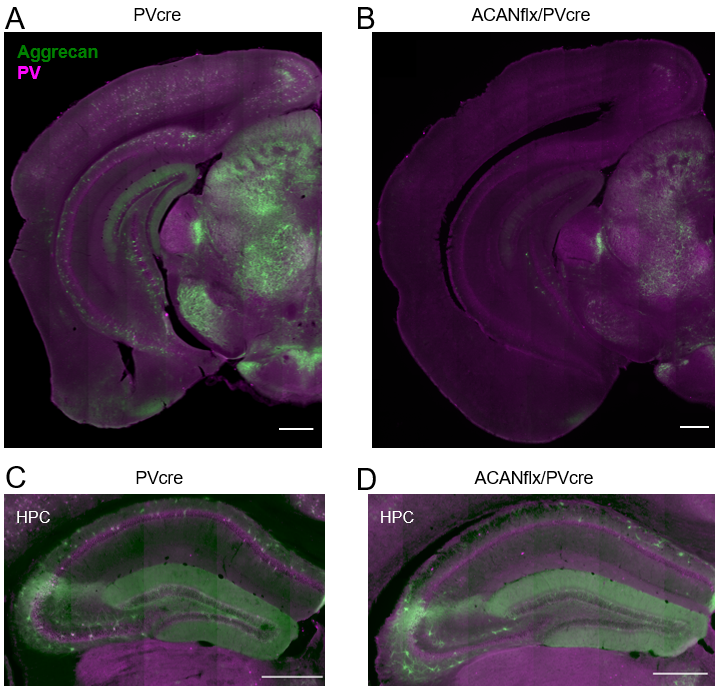


**S1. PV-targeted *Acan* knockout (ACANflx/Pvcre) causes a brain-wide depletion of Aggrecan+ PNNs around PV+ neurons.** *A) Coronal section from a PVcre mouse stained with* Aggrecan *(green) and PV (magenta). B) Coronal section from a ACANflx/Pvcre mouse stained with Aggrecan (green) and PV (magenta). C) Coronal section showing the hippocampus (HPC) of a PVcre mouse stained with Aggrecan (green) and PV (magenta). D) Coronal section showing the hippocampus (HPC) of a ACANflx/PVcre mouse stained with* Aggrecan *(WFA) (green) and PV (magenta). Scale bar = 500 µm.*


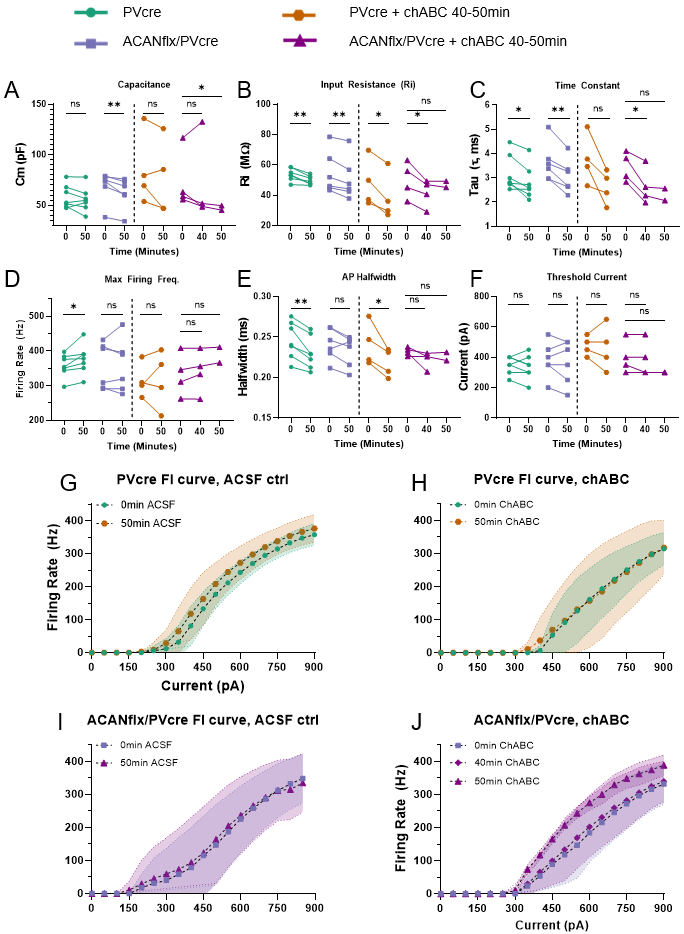


**Figure S2**. **PV+ neurons in acute slices from Pvcre or ACANflx/Pvcre mice treated for 50min with either plain ACSF or ACSF+1U/ml chABC display altered intrinsic electrophysiological and firing properties. *A)*** *Capacitance (CM) is significantly reduced in both ACANflox/PVcre treated with ACSF.* ***B)*** *Input resistance (Ri) is significantly reduced in all groups.* ***C)*** *Time constant is reduced in all groups but PVcre + chABC.* ***D)*** *Max Firing Frequency (Hz) is increased in ACSF-treated PVcre, but not significantly changed in any other group.* ***E)*** *Action potential half-width is reduced in PVcre treated with ACSF and chABC****. F)*** *Threshold current is unchanged in all groups.* ***G-J)*** *FI curves are unaffected by all treatments. All comparisons were made with paired t-tests. PVcre n = 7 cells, 5 animals, ACANflox/PVcre n = 6 cells, 5 animals, PVcre+chABC n = 4 cells, 4 animals, ACANflox/PVcre-40min n = 2 cells, 2 animals, ACANflox/PVcre-50min 4 cells, 3 animals.*

**Figure S3**


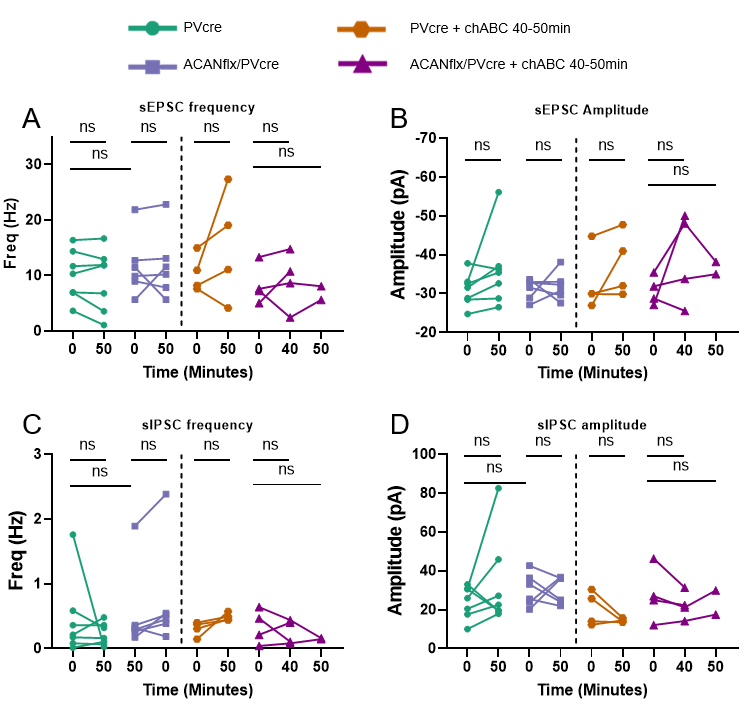


**Figure S3**. **sEPSC and sIPSC frequency and amplitude is unchanged in** **PV+ cells in acute slices from Pvcre or ACANflx/Pvcre mice treated for 50min with either plain ACSF or ACSF+1U/ml chABC*. A-D)*** *No changes were found in sEPSC or sIPSC frequency or amplitude in any treatment groups. All comparisons made with paired t-tests. PVcre n = 7 cells, 5 animals, ACANflox/PVcre n = 6 cells, 5 animals, PVcre+chABC n = 4 cells, 4 animals, ACANflox/PVcre-40min n = 2 cells, 2 animals, ACANflox/PVcre-50min 4 cells, 3 animals.*

**Figure S4**

**
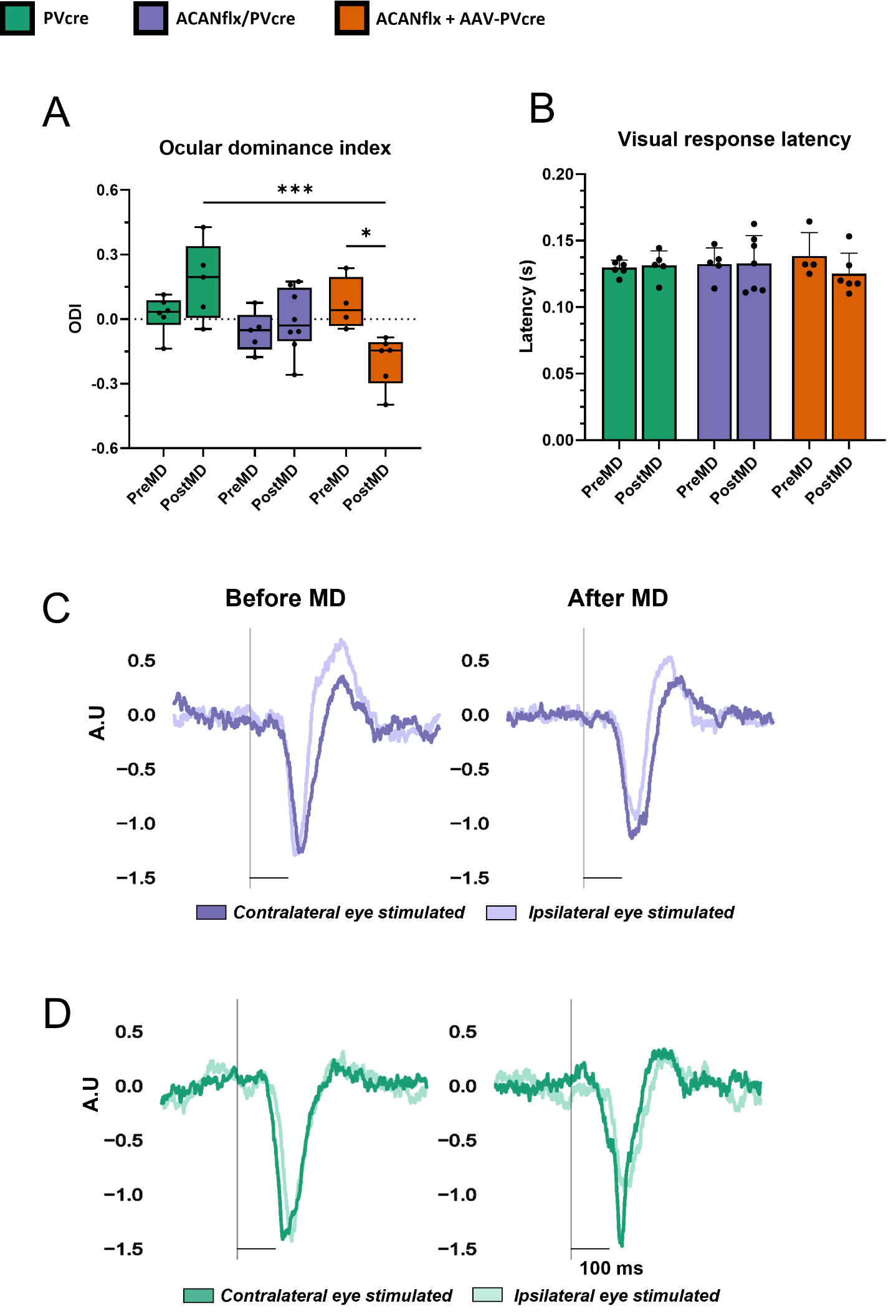
**

**Figure S4: Ocular dominance and visual response latency*.*** *A) Ocular dominance shift was observed in ACANflx+AAV-PVcre when comparing ODI pre- and post-MD (p = 0.044) as well as compared to PVcre controls after MD (p = 0.0008). There were no differences within or between other groups (Pre- vs. post: PVcre p = 0.39, ACANflx/PVcre p = 0.98. Pre-MD: PVcre vs. ACANflx/PVcre p = 0.90, ACANflx/PVcre vs. ACANflx+AAV-PVcre p = 0.69. Post-MD: PVcre vs. ACANflx/PVcre p = 0.16, ACANflx/PVcre vs. ACANflx+AAV-PVcre p = 0.12). B) Latency of LFP response to visual stimulus. No differences were observed. C,D) Average normalized LFP responses of C) all ACANflx/PVcre and D) all PVcre mice. A,B,C: Pre-MD: PVcre N = 6, ACANflx/PVcre N = 5, ACANflx+AAV-PVcre N = 4. Post-MD: PVcre N = 5, ACANflx/PVcre N = 8, ACANflx + AAV-PVcre N = 6. A,B: ANOVA with Tukey’s multiple comparisons test. Fig A and B: data shown as mean ± SD.*

**Figure S5**


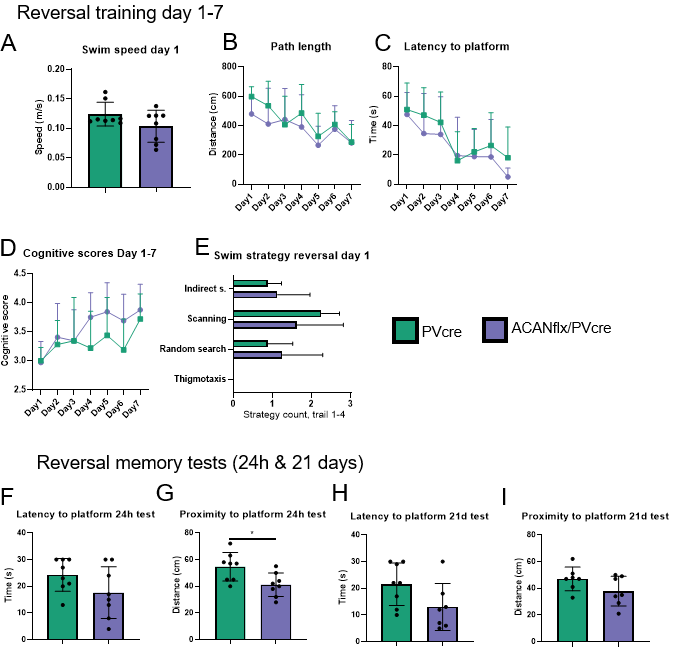


**Figure S5 Reversal training and testing in Morris water maze** *A) Swim speed on day one. B) Path length (cm) during reversal training day 1-7 (Row factor (days of training), p-value = 0.001). C) Latency to platform (s) during reversal training day 1-7 (Row factor (days of training), p-value < 0.0001). D) Cognitive scores given from day 1-7 of reversal training. E) Swim strategy day 1 of reversal training. No occurrences of thigmotaxis. F) Latency (s) to platform during 24 hour memory test G) Proximity (cm) to platform during 24 hour test (mean proximity (cm) PVcre 54.63 cm ± 9.9, ACANflox/PVcre 41.13 cm ± 8.3, p-value = 0.02). H) Latency (s) to platform during the 21 day test. I) Proximity (cm) to platform. Repeated measures ANOVA with Sidak’s multiple comparisons test used for A, B and D. Unpaired Student's t-test used for C and F-I. Data shown as mean ± SD. Each dot represents one animal. PVcre (green) n= 8, ACANflox/PVcre (purple) n= 8.*

**Figure S6**


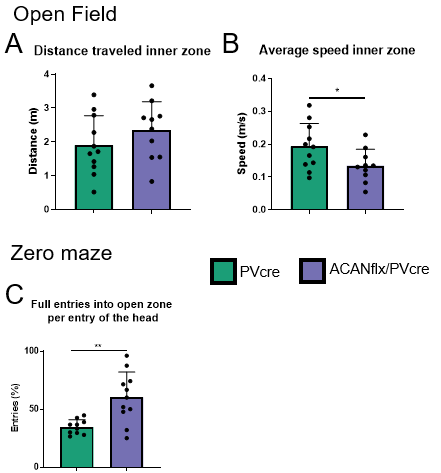


**Figure S6.  ACANflx/Pvcre demonstrates a lower level of anxiety and risk assessment behavior in the open field and zero maze compared to controls**. *Open field A) Distance (m) traveled in the inner zone. B) Average speed (m/s) in the inner zone (PVcre 0.19 m/s ±  0.07, AcanKO 0.13 m/s ± 0.05, p-value = 0.04). Zero maze. C) Likelihood (%) of entering the whole body in the open zone after entering the head (PVcre 34.68% ± 5.93, AcanKO 60.24% ± 20.81, p-value = 0.002). Unpaired Student’s t-test. Data shown as mean ± SD. Each dot represents one animal. PVcre (green) n= 10, AcanKO (purple) n = 11.*

**Table S1, qPCR primers.**

| **Gene name** | **Forward 5’-** | **Reverse 5’-** |
| --- | --- | --- |
| ACAN (exon 4) | GCTTGCCTACAGAACAGCGCCA | GGGGCGTGTGGATGGGGTATCT |
| BCAN | CTCGGCGGCTATGAGCAGTGTG | CAGGCCTCTCGTGGGTTCTGGA |
| VCAN | TGGCCCAGAACGGAAATATCA | ACTAGCCCGGAGTTTGACCAT |
| NCAN | ACGCCTACTGCTTCCGAGCTCA | GGAGGCCCCTCTGCTGACACAA |
| TNR | CCTTGCTGCGAGACCAGTGCAA | TAAAGTTGCCATGGCCGCTGCA |
| SEMA3A | GGCTGGTTCACTGGGATTG | CCGTTTGCATAGTTTGCTCTGG |
| GAPDH | AGGTCGGTGTGAACGGATTTG | TGTAGACCATGTAGTTGAGGTCA |
| PPIA | TCCGACTGTGGACAGCTCTA | ATTGCGAGCAGATGGGGTAG |
